# Fine-scale partitioning of genomic variation among recruits in an exploited fishery: causes and consequences

**DOI:** 10.1038/srep36095

**Published:** 2016-10-26

**Authors:** Jonathan B. Puritz, John R. Gold, David S. Portnoy

**Affiliations:** 1 Marine Genomics Laboratory Department of Life Sciences Texas A&M University-Corpus Christi Corpus Christi, TX 78412, USA; 2 Marine Science Center Northeastern University Nahant, MA 01908, USA.

## Abstract

Conservation and management of exploited species depends on accurate knowledge of how genetic variation is partitioned across a fishery, especially as it relates to recruitment. Using double-digest restriction-site associated DNA sequencing, we surveyed variation in 7,382 single nucleotide polymorphisms (SNPs) in red snapper (*Lutjanus campechanus*) young-of-the-year (YOY) sampled at six localities and in adults sampled at two localities in the northern Gulf of Mexico. Significant genetic heterogeneity was detected between the two adult samples, separated by ~600 km, and at spatial scales less than five kilometers among samples of  YOY. Genetic differences between YOY samples and between YOY samples and adult samples were not associated with geographic distance, and a genome scan revealed no evidence of loci under selection. Estimates of the effective number of breeders, allelic richness, and relatedness within YOY samples were not consistent with sweepstakes recruitment. Instead, the data demonstrate, at least within one recruitment season, that multiple pulses of recruits originate from distinct groups of spawning adults, even at small spatial scales. For exploited species with this type of recruitment pattern, protection of spawning adults over wide geographic areas may be critical for ensuring productivity and stability of the fishery by maintaining larval supply and connectivity.

Genetic heterogeneity observed among recruits in marine organisms (genetic patchiness) is thought to be generated by a number of different processes, including: 1) variance in reproductive success or sweepstakes recruitment in extreme cases, 2) natural selection operating on early life stages, and/or 3) contribution from localized subdivided groups of breeders^1^. Understanding which of these processes drives genetic patchiness is important for restoration and management of exploited species because the presence of independent groups of breeders must be accounted for to properly to characterize recruitment dynamics and maintain neutral and adaptive variation^2^. Further, conservation efforts, such as planning for marine protected areas (MPAs), require an assessment of whether exploited populations are sustained by few or many sources of recruitment^3^,^4^.

Red snapper (*Lutjanus campechanus*) is the target of the largest combined recreational and commercial fisheries in the United States^5^. The fishery has experienced high levels of exploitation since the last half of the 20^th^ century when abundance decreased by 90%^6^, and has been overfished since at least 1980^5^. Previous genetic work has reported genetic homogeneity among adults sampled across the northern Gulf of Mexico^7^,^8^,^9^,^10^,^11^,^12^. However, regional differences in effective population size^13^ and spatial autocorrelation of microsatellite genotypes in young-of-the-year (YOY) recruits at scales between 50–100 km^14^ have also been documented.

Results of prior genetic studies of red snapper are consistent with a metapopulation-type structure^11^,^13^,^14^ defined as semi- independent subpopulations that interact creating some level of demographic interdependence^4^. However, because of constraints of sampling and methodology, neither sweepstakes recruitment nor selection could be assessed. Here, we use double-digest restriction-site associated DNA sequencing (ddRAD)^15^,^16^ to characterize patterns of genomic variation within and between samples of YOY red snapper and two samples of potential breeders, while simultaneously assessing whether the data are consistent sweepstakes recruitment, selection operating during early life history, and/or sub-structured breeding adults.

## Methods

**Figure 1:**
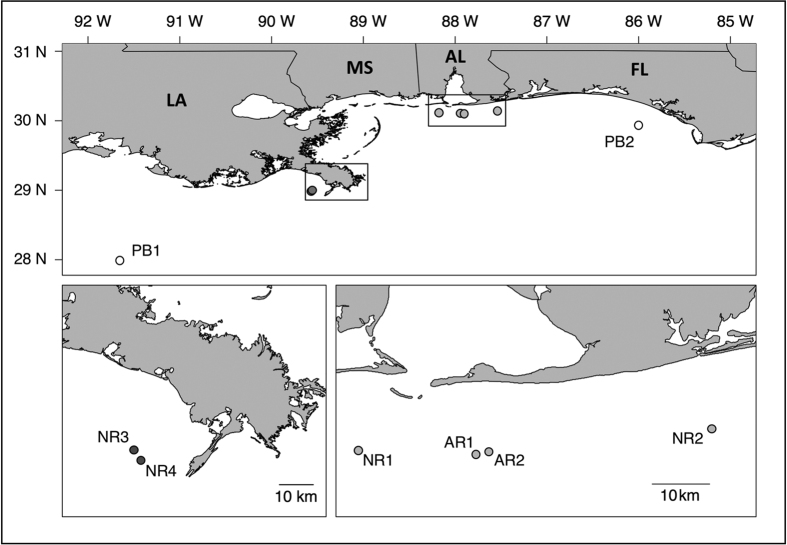
Map of red snapper (*Lutjanus campechanus*) sampling locations. The two lower panels are insets of the top panel at smaller spatial scales. Adult samples are represented with white circles, young of the year (YOY) from Louisiana in dark grey circles, and YOY from Alabama in light grey. Maps were created using R version 3.2.2 (“R: A Language and Environment for Statistical Computing, R Core Team, R Foundation for Statistical Computing, Vienna, Austria (2016) https://www.R-project.org”).

Samples of YOY red snapper were collected during the late fall of 2012 and early spring of 2013 from six, near-shore reefs and represent a single recruiting cohort. Reefs were a combination of natural patch reef (NR) and small experimental reefs (AR). Adults sample were collected between 2011 and 2013 from two localities bracketing the YOY samples (Fig. 1). Metadata for each individual is given in Supplementary Table 1. ddRAD libraries were prepared following Portnoy *et al*.^17^; details may be found in Supplementary Methods. Assembly, mapping, and SNP genotyping employed the *dDocent* pipeline^17^. The initial dataset consisted of 339,032 variant SNP loci across 38,122 fragments. SNPs were extensively filtered and the final dataset consisted of 7,382 SNPs in 205 individuals. SNPs were organized into 2,076 haplotypes, using a custom script. All bioinformatic code has been commented and saved at (https://github.com/jpuritz/Puritz.et.al.2016.Scientific.Reports), raw sequence data has been deposited in the Short Read Archive of NCBI (BioProject PRJNA329407), and Raw SNPs, Final SNPs, and haplotype calls can be found on Figshare (Please see Data Accessibility Section).

Bayescan^18^ was used to identify individual outlier loci by assessing fit to different models of selection. The program was run with all default values, with the exception of 30 pilot runs and a thinning interval of 100; significance of outlier loci was determined using a *q*-value which directly corresponded to a false discovery rate of 0.05. Loci with a *q*-value less than 0.1 were removed from other analyses. Homogeneity of haplotype distributions was tested using single-level analysis of molecular variance (Amova), and pairwise *F*_*ST*_ values and Nei’s genetic distance^19^ were calculated using GenoDive^20^ using 10,000 permutations to test for significance. A neighbour-joining (NJ) tree^21^ was created in Mega^22^ using Nei’s genetic distance. The effective number of breeders (*N*_*b*_) was estimated for each YOY sample, using the linkage disequilibrium method^23^ with a 0.05 frequency cutoff, as implemented in NeEstimator^24^. ML-Relate^25^ was used to estimate pairwise relatedness and Heirfstat^26^ was used to estimate rarefied allelic richness. Probability estimates based on multiple comparisons were corrected using a false discovery rate (FDR) procedure^27^.

## Results

**Figure 2:**
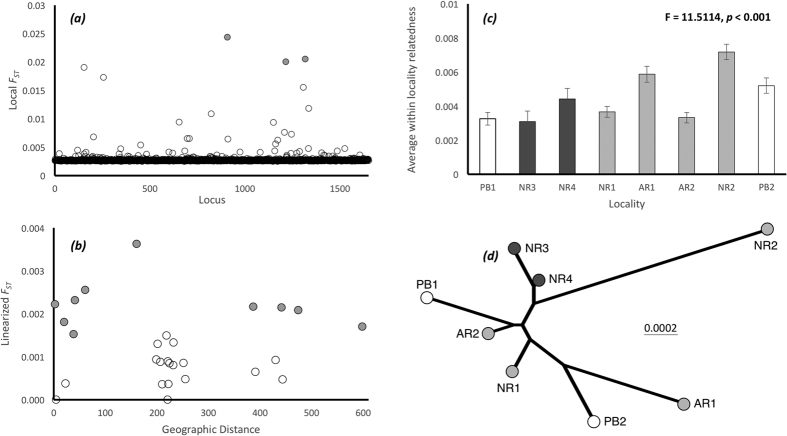
Results of genetic analyses. (**a**) *F*_*ST*_ values generated during outlier detection. Three loci were not outliers (0.1 > q > 0.05), but were removed to ensure neutrality (shaded in grey). (**b**) Pairwise *F*_*ST*_ values (significant values are grey) plotted against geographic distance. (**c**) Average within-locality relatedness; (**d**) Neighbour-joining tree generated from pairwise estimates of Nei’s genetic distance. In (**c,d**) locality geography and sample type are differentiated by different shading: Adult samples have no shading; YOY samples near Louisiana are in dark grey, and YOY samples near Alabama are shaded in light grey.

Genetic differentiation among samples was low but statistically significant (global *F*_*ST*_ = 0.001; *P* = 0.001); no outlier loci were detected (Fig. 2a), though three borderline loci were removed from further analysis. Significant heterogeneity was found between adult samples, between a subset of YOY samples, and between some adult and YOY comparisons (Table 1, Fig. 1b). Inspection of estimates of pairwise *F*_*ST*_ (Table 1) revealed that two YOY samples AR1 (4 of 7 significant comparisons) and NR2 (5 of 7 significant comparisons) were the most differentiated. No relationship between genetic distance and geography was evident (Fig. 2b); sites AR1 and AR2, for example, are separated by ~2 km, yet differ significantly in allele frequencies (*F*_*ST*_ = 0.002; *P* = 0.001), whereas sites PB1 and AR2 are ~443 km apart and genetically homogeneous (*F*_*ST*_ = 0.0005; *P* = 0.144). No coherent geographic clustering of YOY localities was identified (Fig. 2c) and genetic differences were not related to artificial vs natural reef habitat.

Average, within-site pairwise relatedness differed significantly among localities but was low (ranging from 0.003 to 0.007, Fig. 2c), and the magnitude of average relatedness was not different between larval and adult samples. Estimates of *N*_*b*_ for YOY samples ranged between 1,281 and 59,110, with 3 of 5 upper confidence intervals at infinity (Table 2). Estimates of *N*_*b*_ for sites AR1 and NR2 were small in comparison with other sites and a single half-sibship was detected in both localities. Removal of these potentially related individuals changed estimates of *N*_*b*_ to 5,514 (CI: 2,578- ∞) and 3,060 (CI: 1,899-7,847), respectively. Genetic diversity, measured by rarefied allelic richness, did not differ significantly among localities (*F* = 1.0807, *P* > 0.3726).

## Discussion

Genomic analyses revealed small-scale, spatial genetic heterogeneity among YOY red snapper sampled at different localities, providing evidence of independent groups of breeding individuals. The observed pattern of diversity across red snapper recruits is likely the result of some form of spatial genetic heterogeneity across breeding adults, and not driven by other processes known to generate genetic patchiness such as selection, sweepstakes recruitment, and oceanography (reviewed in Toonen and Grosberg^1^). Instead, the observed significant differences between adult populations, large variances in *N*_*b*_, and inconsistent genetic similarities between adult and YOY localities are indicative of groups of independent breeding adults whose contribution to the next generation varies spatially, and likely temporally. The interactions of such groups, through larval dispersal and other processes, may explain results in previous studies that were described as metapopulation-like structure^13^,^14^,^11^.

The absence of significant *F*_*ST*_ outlier loci indicates that divergent selection is not likely driving the patterns of genomic diversity in red snapper recruits. However, even with over 2,000 loci genotyped, much of the red snapper genome remains unsampled, making it difficult to reject the role of selection outright. Moreover, if selection is acting on life stages beyond newly recruited juveniles but prior to spawning, our sampling would not have been adequate to detect it. This could potentially play an important role in shaping the genetic structure of red snapper in the Gulf of Mexico and requires further study.

Multiple lines of evidence strongly indicate that the observed genetic patchiness is not congruent with sweepstakes recruitment. Estimates of *N*_*b*_ were large for several of the YOY populations which would not be expected if a small number of breeders contributed to a recruitment event. While single estimates of *N*_*b*_ can be influenced by the overall effective population size, if subpopulations are weakly differentiated^28^, these numbers are likely too large to be driven by a few successful individuals. Two half-sibships were also detected in the data set, but overall estimates of average pairwise relatedness of YOY samples were low and within-sample estimates of genomic diversity were similar between adult and YOY populations, suggesting that extreme reproductive variance of individuals is not driving the observed pattern.

Heterogeneity between samples of adult red snapper separated by ~500 km was detected indicating independent groups of reproductive individuals. The observed heterogeneity is in contrast to prior genetic studies that have not detected genetic differences across spatial scales of ~1,600 km^7^,^8^,^9^,^10^,^11^,^12^,^13^,^14^, and is likely attributable to use of >2,000 loci in the current analysis. Estimated genomic differences between YOY samples showed no spatial pattern with respect to one another nor was there a spatial pattern with regards to genomic differences/similarity between YOY samples and the adult samples which represent potential breeders. Collectively, this suggests that sources (breeders) for juveniles have the potential to contribute recruits across a wide geographic area, in this case encompassing the entire sampling of the current study (~600 km), which is not surprising given that red snapper have a pelagic larval duration of 3–4 weeks^29^.

These results are consistent with a metapopulation-type model similar to the one proposed by Kritzer and Sale^4^; where the metapopulation is viewed as a network of partially closed subpopulations that interact demographically by sporadic pulses of recruits. Results from this study, change the potential scale over which the dynamic could produce genetic heterogeneity with distinct groups of breeders independently contributing to recruitment at very small spatial scales (< 5 km). It is important to note that the YOY samples represent a single cohort and that the observed pattern with respect to a given sampling locality may be ephemeral. However, YOY cohorts sampled in 2004 and 2005 from several localities in the Gulf of Mexico did not differ significantly in microsatellite allele distributions^14^, suggesting temporal stability can and does exist in some localities.

Metapopulation dynamics in an exploited species could potentially have important management implications. First, equal exploitation of all independent groups within a metapopulation may lead to a reduction in both productivity and temporal stability^30^. This may be particularly problematic if sink-source dynamics exist among subpopulations and are not well known^31^. Further, older fish may have greater reproductive output than younger fish^32^, and for exploited species such as red snapper, where the older age class have been removed^33^, larval supply could be constrained. A reduction of larval supply across semi-independent demes, for example, could reduce connectivity, enhance genetic drift, and erode genetic diversity^34^; a dynamic that is congruent with findings from a recent meta-analysis^35^.

Given the potential for long distance larval dispersal in red snapper, further work with increased temporal and spatial sampling will be required to assess how many reproductive units might be present and whether or not these units consistently and predictably contribute recruits to specific areas. Genomic monitoring of fisheries species across different age classes at biologically meaningful spatial and temporal scales will be important for informing management and designating essential fish habitat, as multiple smaller refuges spread across a large geographic distance may be more effective in preserving natural recruitment dynamics, for species with metapopulation structure, than fewer larger reserves^3^,^4^.

## Data Accessibility

Bioinformatic code (https://github.com/jpuritz/Puritz.et.al.2016.Scientific.Reports)

Raw sequence data (SRA: BioProject PRJNA329407)

Raw SNP calls (https://figshare.com/articles/TotalRawSNPs_vcf_gz/3490226)

Filtered SNPs (https://figshare.com/articles/Final_filtered_SNPs_vcf/3490232)

Final haplotype calls (https://figshare.com/articles/Final_Haps_gen/3490229)

## Additional Information

**How to cite this article**: Puritz, J. B. *et al*. Fine-scale partitioning of genomic variation among recruits in an exploited fishery: causes and consequences. *Sci. Rep.* **6**, 36095; doi: 10.1038/srep36095 (2016).

**Publisher’s note:** Springer Nature remains neutral with regard to jurisdictional claims in published maps and institutional affiliations.

## Supplementary Material

Supplementary Information

## Figures and Tables

**Table 1:** Pairwise *F*_*ST*_ values: significant values after FDR correction are bolded.

	PB1	NR3	NR4	NR1	AR1	AR2	NR2
PB1							
NR3	0.0008	—					
NR4	0.0013	−0.0006	—				
NR1	0.0009	0.0004	0.0009	—			
AR1	**0.0021**	0.0004	0.0015	**0.0018**	—		
AR2	0.0005	0.0008	−0.0002	0.0004	**0.0022**	—	
NR2	**0.0021**	0.0005	0.0009	**0.0025**	**0.0023**	**0.0015**	—
PB2	**0.0017**	0.0006	**0.0022**	0.0009	0.0013	0.0009	**0.0036**

**Table 2:** Estimates of the effective number of breeders (*N*_*b*_) at each locality.

Locality	*N*_*b*_	Lower 95%	Upper 95%
NR3/NR4	11,616	5,293	∞
NR1	10,540	5,025	∞
AR1	1,281	1,022	1,714
AR2	59,110	8,819	∞
NR2	1,663	1,254	2,466

Samples NR3 and NR4 were combined because no significant genetic difference was detected between them.
