# Supplementary material for: Fine-scale partitioning of genomic variation among recruits in an exploited fishery: causes and consequences
Source: Sci Rep. 2016 Oct 26;6:36095. doi: 10.1038/srep36095 (PMC5080595; doi:10.1038/srep36095)
Supplement: Supplementary Information [file srep36095-s1.doc]

**Supplementary Information**

**for**

**“Fine-scale partitioning of genomic variation among recruits in an exploited fishery: causes and consequences”**

Jonathan B. Puritz*^1,2^, John R. Gold^1^, and David S. Portnoy^1^

^1^Marine Genomics Laboratory

Department of Life Sciences

Texas A&M University-Corpus Christi

Corpus Christi, TX 78412

^2^Marine Science Center

Northeastern University

Nahant, MA 01908

*To whom correspondence should be addressed: jpuritz@gmail.com

Keywords: population genomics, recruitment variability, ddRAD, fisheries

**Supplemental Methods:**

*ddRADseq-* DNA was extracted using Mag-Bind Tissue DNA kits (Omega Bio-Tek) and digested with *Eco*RI and *Msp*I. A barcoded adapter was ligated to *Eco*RI restriction sites and a common adapter was ligated to *Msp*I restriction sites, using equimolar quantities of each digested sample. Samples were then pooled into four ‘index’ libraries consisting of ~30 individuals each and size selected using a Pippin Prep DNA size selection system (Sage Science Inc.). Fragments were selected using a mean size of 375 bp, with a ‘tight’ selection window (± 37 bp). Illumina flow-cell adapter sequences and index-specific identifiers were added to each index library, using 12 cycles of PCR. Sequencing was spread out over three lanes of Illumina sequencing.

*Bioinformatic Analysis*

The *dDocent* pipeline (version 2.1; [1]) was used for *de novo* assembly, read trimming, read mapping, and SNP genotyping. After examining multiple iterations, assembly parameters of k1=4, k2=6, and clustering similarity equalled 90%. For read mapping, the mismatch parameter was lowered from 4 to 3, and the gap opening penalty was lowered from 6 to 5. Raw variant calls were subjected to several filtering steps to reduce false positives. All bioinformatic code has been commented and saved at (<https://github.com/jpuritz/Puritz.et.al.2016.Scientific.Reports)>. Raw variants were filtered sequentially via VCFtools (<https://vcftools.github.io/index.html>; [2]) or custom bash scripts, using the following steps:

1. All genotypes with less than 5 reads were changed to missing.
2. Loci were removed that had a minor allele frequency of less than 1% and a call rate of less than 50%.
3. 36 individuals (out of 241) were removed for having more than 42% missing data, using the script filter_missing_ind.sh (<https://github.com/jpuritz/dDocent/blob/master/scripts/filter_missing_ind.sh)>.
4. Loci were then removed that were not called in 75% of individuals in any one population using the script pop_missing_filter.sh (https://github.com/jpuritz/dDocent/blob/master/scripts/pop_missing_filter.sh)
5. Loci called in less than 90.0% of individuals overall and with a minor allele frequency of less than 1% were removed.

After this point, variant calls were filtered using the script (dDocent_filters; https://github.com/jpuritz/dDocent/blob/master/scripts/dDocent_filters) that utilizes vcflib (https://github.com/ekg/vcflib) and VCFtools (https://vcftools.github.io/index.html) to filter loci based on FreeBayes INFO criteria and depth:

1. Loci were removed if the average allele balance at heterozygous genotypes was less than 25% (i.e., if a genotype had 100X coverage, there would have to be an average of 28 or more reads from the alternate allele across all heterozygous genotypes). Additionally, if the quality sum of the reference or alternate allele was 0, the locus was removed. This removes sites that have a large portion of spurious heterozygous genotype calls.
2. Loci were then removed if the quality score was less than half of the total depth. With FreeBayes, excessive depth can give inflated quality scores.
3. Loci were removed if the ratio between the mean mapping quality of the alternate and reference allele was less than 0.9 or more than 1.05.
4. Loci were then removed if the majority of reads did not come from only one read orientation. Our insert size was much larger than our PE read lengths, so true RAD loci should not have forward and reverse reads that overlap.
5. Loci also were removed based on the status of properly paired reads. True variants should have reads coming from all properly paired reads, or only from reads that are not properly paired (some RAD loci do not assemble well for the PE read, leaving only forward reads). However, false variants tend to have properly paired reference reads and not properly paired alternate reads. Loci were retained if more than 0.05% of reference reads were properly paired and less than 0.05% of alternate reads were properly paired and vice versa.
6. Of the remaining loci, the average depth (and standard deviation) across all individuals was calculated. Loci that have a depth greater than the average depth plus on SD are removed if the quality score is less than 2 times the depth. This filter is based off results reported by Li [3].
7. Only loci that were in the bottom 90% of mean depth (less than 124) were kept to remove any possible paralogs or repetitive genomic regions.

Variant calls were then decomposed into SNP and INDEL calls, using vcflib; INDELS were then removed with VCFtools to produce a VCF file of SNP only calls. SNPs were filtered based on locality-based tests of Hardy Weinberg equilibrium. Loci were removed that had a *P* value of less than 0.001 in at least 50% of the populations, using the script filter_hwe_by_pop.pl (<https://github.com/jpuritz/dDocent/blob/master/scripts/filter_hwe_by_pop.pl)>. SNPs were then filtered to only include loci with 2 alleles using VCFtools. SNPs were then haplotyped using the script rad_haplotyper.pl to search for loci that might be paralogous or have genotyping errors. Loci that had more than 5 individuals marked as paralogous and more than 30 individuals marked for potential genotyping errors were removed using the script remove.bad.hap.loci.sh (<https://github.com/jpuritz/dDocent/blob/master/scripts/remove.bad.hap.loci.sh)>.

To test for loci that were potentially biased during library prep, samples from locality NR3 were spread across two libraries and lanes of Illumina sequencing. These individuals from the same locality were separated into pseudo-populations based on library and loci were examined for FST outliers using the program Lositan (<http://popgen.net/soft/lositan/>; [4,5]). Loci found to have a greater than 99.9% probability of being an outlier and an FST value greater than 0.1 were removed from the data set, as they are likely biased.

SNPs were then filtered for a minor allele frequency of 5% and then converted into a BayeScan [6] input file. BayeScan was run using default parameters with the exception of changing the thinning interval to 100 and the number of pilot runs to 30. Loci with a q-value below 0.05 were visually examined across several samples to look for any potential source of library bias, such as systematic differences in trimming, cut site polymorphism, length variants, etc. Loci identified as problematic were removed.

After this final step, loci with more than 2 alleles were removed, and then remaining loci were haplotyped and converted into a final GENEPOP file for analysis.

**Supplemental References:**

1. Puritz, J. B., Hollenbeck, C. M. & Gold, J. R. 2014 dDocent: a RADseq, variant-calling pipeline designed for population genomics of non-model organisms. *PeerJ* **2**, e431. (doi:10.7717/peerj.431)

2. Danecek, P. et al. 2011 The variant call format and VCFtools. *Bioinformatics* **27**, 2156–8. (doi:10.1093/bioinformatics/btr330)

3. Li, H. 2014 Toward better understanding of artifacts in variant calling from high-coverage samples. *Bioinformatics* **30**, 2843–2851. (doi:10.1093/bioinformatics/btu356)

4. Antao, T., Lopes, A., Lopes, R. J., Beja-Pereira, A. & Luikart, G. 2008 LOSITAN: a workbench to detect molecular adaptation based on a Fst-outlier method. *BMC Bioinformatics* **9**, 323. (doi:10.1186/1471-2105-9-323)

5. Beaumont, M. A. & Nichols, R. A. 1996 Evaluating loci for use in the genetic analysis of population structure. *Proc. Biol. Sci.* **263**, 1619–1626. (doi:10.1098/rspb.1996.0237)

6. Foll, M. & Gaggiotti, O. 2008 A genome-scan method to identify selected loci appropriate for both dominant and codominant markers: a Bayesian perspective. *Genetics* **180**, 977–93. (doi:10.1534/genetics.108.092221)

7. Nei, M. 1972 Genetic distance between populations. *Am. Nat.* **106**, 283–292. (doi:10.2307/2678832)

8. Meirmans, P. G. & Van Tienderen, P. H. 2004 Genotype and Genodive: Two Programs for the Analysis of Genetic Diversity of Asexual Organisms. *Mol. Ecol. Notes* **4**, 792–794. (doi:10.1111/j.1471-8286.2004.00770.x)

9. Saitou, N. & Nei, M. 1987 The Neighbor-joining Method: A New Method for Reconstructing Phylogenetic Trees. *Mol. Biol. Evol.* **4**, 406–425. (doi:citeulike-article-id:93683)

10. Tamura, K., Stecher, G., Peterson, D., Filipski, A. & Kumar, S. 2013 MEGA6: Molecular evolutionary genetics analysis version 6.0. *Mol. Biol. Evol.* **30**, 2725–2729. (doi:10.1093/molbev/mst197)

11. Benjamini, Y. & Hochberg, Y. 1995 Controlling the false discovery rate: a practical and powerful approach to multiple testing. *J. R. Stat. Soc.* **57**, 289–300. (doi:10.2307/2346101)

**Table S1**

| **Location Code** | **Sample Name** | **Latitude** | **Longitude** | **SL (mm)** | **TL**  **(mm)** | **Weight (g)** | **Date Collected** | **Included after filtering** | **Age Class** |
| --- | --- | --- | --- | --- | --- | --- | --- | --- | --- |
| NR3 | ELA_001 | 29° 00.05' N | 89° 34.99' W | 120 | 162 | - | 4/29/13 | YES | YOY |
| NR3 | ELA_002 | 29° 00.05' N | 89° 34.99' W | 106 | 145 | - | 4/29/13 | YES | YOY |
| NR3 | ELA_003 | 29° 00.05' N | 89° 34.99' W | 95 | 129 | - | 4/29/13 | YES | YOY |
| NR3 | ELA_004 | 29° 00.05' N | 89° 34.99' W | 101 | 137 | - | 4/29/13 | YES | YOY |
| NR3 | ELA_005 | 29° 00.05' N | 89° 34.99' W | 113 | 152 | - | 4/29/13 | YES | YOY |
| NR3 | ELA_006 | 29° 00.05' N | 89° 34.99' W | 95 | 125 | - | 4/29/13 | YES | YOY |
| NR3 | ELA_007 | 29° 00.05' N | 89° 34.99' W | 77 | 105 | - | 4/29/13 | YES | YOY |
| NR3 | ELA_008 | 29° 00.05' N | 89° 34.99' W | 83 | 112 | - | 4/29/13 | YES | YOY |
| NR3 | ELA_009 | 29° 00.05' N | 89° 34.99' W | 87 | 102 | - | 4/29/13 | YES | YOY |
| NR3 | ELA_010 | 29° 00.05' N | 89° 34.99' W | 89 | 116 | - | 4/29/13 | YES | YOY |
| NR3 | ELA_011 | 29° 00.05' N | 89° 34.99' W | 85 | 114 | - | 4/29/13 | YES | YOY |
| NR3 | ELA_012 | 29° 00.05' N | 89° 34.99' W | 84 | 112 | - | 4/29/13 | YES | YOY |
| NR3 | ELA_013 | 29° 00.05' N | 89° 34.99' W | 83 | 109 | - | 4/29/13 | YES | YOY |
| NR3 | ELA_014 | 29° 00.05' N | 89° 34.99' W | 82 | 113 | - | 4/29/13 | YES | YOY |
| NR3 | ELA_015 | 29° 00.05' N | 89° 34.99' W | 84 | 115 | - | 4/29/13 | YES | YOY |
| NR3 | ELA_016 | 29° 00.05' N | 89° 34.99' W | 93 | 123 | - | 4/29/13 | YES | YOY |
| NR3 | ELA_017 | 29° 00.05' N | 89° 34.99' W | 94 | 124 | - | 4/29/13 | YES | YOY |
| NR3 | ELA_018 | 29° 00.05' N | 89° 34.99' W | 114 | 152 | - | 4/29/13 | YES | YOY |
| NR4 | ELA_019 | 29° 00.05' N | 89° 34.99' W | 143 | 187 | - | 4/29/13 | YES | YOY |
| NR4 | ELA_020 | 29° 00.05' N | 89° 34.99' W | 124 | 161 | - | 4/29/13 | YES | YOY |
| NR4 | ELA_021 | 28° 58.20' N | 89° 33.70' W | 128 | 173 | - | 4/29/13 | YES | YOY |
| NR4 | ELA_022 | 28° 58.20' N | 89° 33.70' W | 135 | 177 | - | 4/29/13 | YES | YOY |
| NR4 | ELA_023 | 28° 58.20' N | 89° 33.70' W | 134 | 179 | - | 4/29/13 | YES | YOY |
| NR4 | ELA_024 | 28° 58.20' N | 89° 33.70' W | 141 | 188 | - | 4/29/13 | YES | YOY |
| NR4 | ELA_025 | 28° 58.20' N | 89° 33.70' W | 140 | 183 | - | 4/29/13 | YES | YOY |
| NR4 | ELA_026 | 28° 58.20' N | 89° 33.70' W | 135 | 174 | - | 4/29/13 | NO | YOY |
| NR4 | ELA_027 | 28° 58.20' N | 89° 33.70' W | 131 | 167 | - | 4/29/13 | YES | YOY |
| NR4 | ELA_028 | 28° 58.20' N | 89° 33.70' W | 156 | 197 | - | 4/29/13 | NO | YOY |
| NR4 | ELA_029 | 28° 58.20' N | 89° 33.70' W | 128 | 153 | - | 4/29/13 | YES | YOY |
| NR4 | ELA_030 | 28° 58.20' N | 89° 33.70' W | 113 | 142 | - | 4/29/13 | YES | YOY |
| NR4 | ELA_031 | 28° 58.20' N | 89° 33.70' W | 118 | 156 | - | 4/29/13 | YES | YOY |
| NR4 | ELA_032 | 28° 58.20' N | 89° 33.70' W | 143 | 183 | - | 4/29/13 | YES | YOY |
| NR4 | ELA_033 | 28° 58.20' N | 89° 33.70' W | 106 | 141 | - | 4/29/13 | YES | YOY |
| NR4 | ELA_034 | 28° 58.20' N | 89° 33.70' W | 138 | 176 | - | 4/29/13 | YES | YOY |
| NR4 | ELA_035 | 28° 58.20' N | 89° 33.70' W | 137 | 174 | - | 4/29/13 | YES | YOY |
| NR4 | ELA_036 | 28° 58.20' N | 89° 33.70' W | 108 | 142 | - | 4/29/13 | YES | YOY |
| NR4 | ELA_037 | 28° 58.20' N | 89° 33.70' W | 97 | 124 | - | 4/29/13 | YES | YOY |
| NR4 | ELA_038 | 28° 58.20' N | 89° 33.70' W | 127 | 163 | - | 4/29/13 | NO | YOY |
| NR4 | ELA_039 | 28° 58.20' N | 89° 33.70' W | 150 | 189 | - | 4/29/13 | YES | YOY |
| AR1 | AR_001 | 30° 6.00' N | 87° 57.00' W | 85 | 107 | 20 | 11/15/12 | YES | YOY |
| AR1 | AR_003 | 30° 6.00' N | 87° 57.00' W | 89 | 112 | 22 | 11/15/12 | YES | YOY |
| AR1 | AR_004 | 30° 6.00' N | 87° 57.00' W | 119 | 148 | 48 | 11/15/12 | YES | YOY |
| AR1 | AR_005 | 30° 6.00' N | 87° 57.00' W | 105 | 135 | 40 | 11/15/12 | YES | YOY |
| AR1 | AR_008 | 30° 6.00' N | 87° 57.00' W | 128 | 163 | 80 | 11/15/12 | YES | YOY |
| AR1 | AR_010 | 30° 6.00' N | 87° 57.00' W | 105 | 133 | 38 | 11/15/12 | YES | YOY |
| AR1 | AR_012 | 30° 6.00' N | 87° 57.00' W | 129 | 162 | 68 | 11/15/12 | YES | YOY |
| AR1 | AR_014 | 30° 6.00' N | 87° 57.00' W | 132 | 167 | 76 | 11/15/12 | YES | YOY |
| AR1 | AR_015 | 30° 6.00' N | 87° 57.00' W | 102 | 130 | 36 | 11/15/12 | YES | YOY |
| AR1 | AR_016 | 30° 6.00' N | 87° 57.00' W | 112 | 143 | 46 | 11/15/12 | YES | YOY |
| AR1 | AR_017 | 30° 6.00' N | 87° 57.00' W | 119 | 154 | 58 | 11/15/12 | YES | YOY |
| AR1 | AR_019 | 30° 6.00' N | 87° 57.00' W | 73 | 93 | 14 | 11/15/12 | YES | YOY |
| AR1 | AR_020 | 30° 6.00' N | 87° 57.00' W | 106 | 136 | 40 | 11/15/12 | YES | YOY |
| AR1 | AR_023 | 30° 6.00' N | 87° 57.00' W | 103 | 131 | 40 | 11/15/12 | YES | YOY |
| AR1 | AR_024 | 30° 6.00' N | 87° 57.00' W | 126 | 160 | 64 | 11/15/12 | YES | YOY |
| AR1 | AR_025 | 30° 6.00' N | 87° 57.00' W | 116 | 150 | 54 | 11/15/12 | YES | YOY |
| AR1 | AR_026 | 30° 6.00' N | 87° 57.00' W | 125 | 160 | 60 | 11/15/12 | YES | YOY |
| AR1 | AR_028 | 30° 6.00' N | 87° 57.00' W | 111 | 143 | 46 | 11/15/12 | YES | YOY |
| AR1 | AR_031 | 30° 6.00' N | 87° 57.00' W | 110 | 137 | 44 | 11/15/12 | YES | YOY |
| AR1 | AR_033 | 30° 6.00' N | 87° 57.00' W | 95 | 119 | 26 | 11/15/12 | YES | YOY |
| AR1 | AR_035 | 30° 6.00' N | 87° 57.00' W | 72 | 93 | 14 | 11/15/12 | YES | YOY |
| AR1 | AR_036 | 30° 6.00' N | 87° 57.00' W | 109 | 140 | 40 | 11/15/12 | YES | YOY |
| AR1 | AR_037 | 30° 6.00' N | 87° 57.00' W | 111 | 139 | 44 | 11/15/12 | YES | YOY |
| AR1 | AR_038 | 30° 6.00' N | 87° 57.00' W | 96 | 124 | 32 | 11/15/12 | YES | YOY |
| AR2 | ARB_001 | 30° 6.30' N | 87° 55.62' W | 132 | 166 | 70.93 | 7/26/13 | YES | YOY |
| AR2 | ARB_002 | 30° 6.30' N | 87° 55.62' W | 182 | 231 | 206 | 7/26/13 | YES | YOY |
| AR2 | ARB_003 | 30° 6.30' N | 87° 55.62' W | 127 | 161 | 67.47 | 7/26/13 | YES | YOY |
| AR2 | ARB_004 | 30° 6.30' N | 87° 55.62' W | 141 | 178 | 89.99 | 7/26/13 | YES | YOY |
| AR2 | ARB_005 | 30° 6.30' N | 87° 55.62' W | 151 | 194 | 118.33 | 7/26/13 | YES | YOY |
| AR2 | ARB_006 | 30° 6.30' N | 87° 55.62' W | 144 | 183 | 95.26 | 7/26/13 | YES | YOY |
| AR2 | ARB_007 | 30° 6.30' N | 87° 55.62' W | 142 | 178 | 98.26 | 7/26/13 | YES | YOY |
| AR2 | ARB_008 | 30° 6.30' N | 87° 55.62' W | 151 | 191 | 108.57 | 7/26/13 | YES | YOY |
| AR2 | ARB_009 | 30° 6.30' N | 87° 55.62' W | 112 | 145 | 45.14 | 7/26/13 | YES | YOY |
| AR2 | ARB_010 | 30° 6.30' N | 87° 55.62' W | 133 | 170 | 74.07 | 7/26/13 | YES | YOY |
| AR2 | ARB_011 | 30° 6.30' N | 87° 55.62' W | 160 | 204 | 138.32 | 7/26/13 | YES | YOY |
| AR2 | ARB_012 | 30° 6.30' N | 87° 55.62' W | 153 | 192 | 113.18 | 7/26/13 | YES | YOY |
| AR2 | ARB_013 | 30° 6.30' N | 87° 55.62' W | 124 | 158 | 64.6 | 7/26/13 | YES | YOY |
| AR2 | ARB_014 | 30° 6.30' N | 87° 55.62' W | 154 | 197 | 120.64 | 7/26/13 | YES | YOY |
| AR2 | ARB_015 | 30° 6.30' N | 87° 55.62' W | 122 | 154 | 62.66 | 7/26/13 | YES | YOY |
| AR2 | ARB_016 | 30° 6.30' N | 87° 55.62' W | 158 | 202 | 127.36 | 7/26/13 | YES | YOY |
| AR2 | ARB_017 | 30° 6.30' N | 87° 55.62' W | 203 | 260 | 288 | 7/26/13 | YES | YOY |
| AR2 | ARB_018 | 30° 6.30' N | 87° 55.62' W | 192 | 246 | 238 | 7/26/13 | YES | YOY |
| AR2 | ARB_019 | 30° 6.30' N | 87° 55.62' W | 159 | 201 | 129.57 | 7/26/13 | YES | YOY |
| AR2 | ARB_020 | 30° 6.30' N | 87° 55.62' W | 118 | 151 | 55.31 | 7/26/13 | YES | YOY |
| AR2 | ARB_021 | 30° 6.30' N | 87° 55.62' W | 203 | 258 | 246 | 7/26/13 | YES | YOY |
| AR2 | ARB_022 | 30° 6.30' N | 87° 55.62' W | 223 | 282 | 352 | 7/26/13 | YES | YOY |
| AR2 | ARB_023 | 30° 6.30' N | 87° 55.62' W | 180 | 225 | 177.07 | 7/26/13 | YES | YOY |
| AR2 | ARB_024 | 30° 6.30' N | 87° 55.62' W | 106 | 136 | 39.8 | 7/26/13 | YES | YOY |
| AR2 | ARB_025 | 30° 6.30' N | 87° 55.62' W | 129 | 161 | 66.46 | 7/26/13 | YES | YOY |
| AR2 | ARB_026 | 30° 6.30' N | 87° 55.62' W | 162 | 211 | 132 | 7/26/13 | YES | YOY |
| AR2 | ARB_027 | 30° 6.30' N | 87° 55.62' W | 127 | 158 | 60.39 | 7/26/13 | YES | YOY |
| AR2 | ARB_028 | 30° 6.30' N | 87° 55.62' W | 141 | 176 | 88.02 | 7/26/13 | YES | YOY |
| AR2 | ARB_029 | 30° 6.30' N | 87° 55.62' W | 179 | 223 | 181.78 | 7/26/13 | YES | YOY |
| AR2 | ARB_030 | 30° 6.30' N | 87° 55.62' W | 202 | 251 | 248 | 7/26/13 | YES | YOY |
| AR2 | ARB_031 | 30° 6.30' N | 87° 55.62' W | 153 | 192 | 106.89 | 7/26/13 | YES | YOY |
| AR2 | ARB_032 | 30° 6.30' N | 87° 55.62' W | 146 | 186 | 97.39 | 7/26/13 | YES | YOY |
| AR2 | ARB_033 | 30° 6.30' N | 87° 55.62' W | 154 | 200 | 112.9 | 7/26/13 | YES | YOY |
| AR2 | ARB_034 | 30° 6.30' N | 87° 55.62' W | 186 | 236 | 206 | 7/26/13 | YES | YOY |
| AR2 | ARB_035 | 30° 6.30' N | 87° 55.62' W | 120 | 151 | 53.65 | 7/26/13 | YES | YOY |
| NR1 | MB_001 | 30° 6.38' N | 88° 9.62' W | - | 69 | 5.1 | 10/9/12 | YES | YOY |
| NR1 | MB_002 | 30° 6.38' N | 88° 9.62' W | - | 99 | 16.2 | 10/9/12 | YES | YOY |
| NR1 | MB_003 | 30° 6.38' N | 88° 9.62' W | - | 102 | 18.9 | 10/9/12 | YES | YOY |
| NR1 | MB_004 | 30° 6.38' N | 88° 9.62' W | - | 124 | 30.5 | 10/9/12 | YES | YOY |
| NR1 | MB_005 | 30° 6.38' N | 88° 9.62' W | - | 90 | 12.1 | 10/9/12 | YES | YOY |
| NR1 | MB_006 | 30° 6.38' N | 88° 9.62' W | - | 110 | 23.7 | 10/9/12 | YES | YOY |
| NR1 | MB_007 | 30° 6.38' N | 88° 9.62' W | - | 73 | 7.2 | 10/9/12 | YES | YOY |
| NR1 | MB_008 | 30° 6.38' N | 88° 9.62' W | - | 123 | 30.7 | 10/9/12 | YES | YOY |
| NR1 | MB_009 | 30° 6.38' N | 88° 9.62' W | - | 118 | 26.6 | 10/9/12 | YES | YOY |
| NR1 | MB_010 | 30° 6.38' N | 88° 9.62' W | - | 117 | 26.3 | 10/9/12 | YES | YOY |
| NR1 | MB_011 | 30° 6.38' N | 88° 9.62' W | - | 114 | 26.1 | 10/9/12 | YES | YOY |
| NR1 | MB_012 | 30° 6.38' N | 88° 9.62' W | - | 117 | 27 | 10/9/12 | YES | YOY |
| NR1 | MB_013 | 30° 6.38' N | 88° 9.62' W | - | 105 | 22 | 10/9/12 | YES | YOY |
| NR1 | MB_014 | 30° 6.38' N | 88° 9.62' W | - | 121 | 28.4 | 10/9/12 | YES | YOY |
| NR1 | MB_015 | 30° 6.38' N | 88° 9.62' W | - | 107 | 20.4 | 10/9/12 | YES | YOY |
| NR1 | MB_016 | 30° 6.38' N | 88° 9.62' W | - | 126 | 33.9 | 10/9/12 | YES | YOY |
| NR1 | MB_017 | 30° 6.38' N | 88° 9.62' W | - | 124 | 31 | 10/9/12 | YES | YOY |
| NR1 | MB_018 | 30° 6.38' N | 88° 9.62' W | - | 107 | 19.6 | 10/9/12 | NO | YOY |
| NR1 | MB_019 | 30° 6.38' N | 88° 9.62' W | - | 120 | 31.3 | 10/9/12 | YES | YOY |
| NR1 | MB_020 | 30° 6.38' N | 88° 9.62' W | - | 130 | 34.9 | 10/9/12 | YES | YOY |
| NR1 | MB_021 | 30° 6.38' N | 88° 9.62' W | - | 121 | 28.2 | 10/9/12 | NO | YOY |
| NR1 | MB_022 | 30° 6.38' N | 88° 9.62' W | - | 107 | 19.9 | 10/9/12 | YES | YOY |
| NR1 | MB_023 | 30° 6.38' N | 88° 9.62' W | - | 90 | 12.6 | 10/9/12 | NO | YOY |
| NR1 | MB_024 | 30° 6.38' N | 88° 9.62' W | - | 75 | 7.7 | 10/9/12 | YES | YOY |
| NR1 | MB_025 | 30° 6.38' N | 88° 9.62' W | - | 125 | 31.7 | 10/9/12 | YES | YOY |
| NR1 | MB_026 | 30° 6.38' N | 88° 9.62' W | - | 69 | 5.5 | 10/9/12 | YES | YOY |
| NR1 | MB_027 | 30° 6.38' N | 88° 9.62' W | - | 119 | 26.5 | 10/9/12 | YES | YOY |
| NR1 | MB_028 | 30° 6.38' N | 88° 9.62' W | - | 103 | 17.1 | 10/9/12 | YES | YOY |
| NR1 | MB_029 | 30° 6.38' N | 88° 9.62' W | - | 147 | 50.5 | 10/9/12 | NO | YOY |
| NR1 | MB_030 | 30° 6.38' N | 88° 9.62' W | - | 112 | 24.7 | 10/9/12 | YES | YOY |
| NR1 | MB_031 | 30° 6.38' N | 88° 9.62' W | - | 113 | 23.4 | 10/9/12 | YES | YOY |
| NR1 | MB_032 | 30° 6.38' N | 88° 9.62' W | - | 68 | 5.3 | 10/9/12 | YES | YOY |
| NR1 | MB_033 | 30° 6.38' N | 88° 9.62' W | - | 111 | 22.8 | 10/9/12 | YES | YOY |
| NR1 | MB_034 | 30° 6.38' N | 88° 9.62' W | - | 102 | 17.8 | 10/9/12 | YES | YOY |
| NR1 | MB_035 | 30° 6.38' N | 88° 9.62' W | - | 103 | 18.2 | 10/9/12 | YES | YOY |
| NR1 | MB_036 | 30° 6.38' N | 88° 9.62' W | - | 153 | 61.8 | 10/9/12 | YES | YOY |
| NR1 | MB_037 | 30° 6.38' N | 88° 9.62' W | - | 125 | 33.3 | 10/9/12 | YES | YOY |
| NR2 | MBB_001 | 30° 8.73' N | 87° 31.74' W | - | 100 | 16 | 10/10/12 | YES | YOY |
| NR2 | MBB_002 | 30° 8.73' N | 87° 31.74' W | - | 58 | 3 | 10/10/12 | YES | YOY |
| NR2 | MBB_003 | 30° 8.73' N | 87° 31.74' W | - | 96 | 16.8 | 10/10/12 | YES | YOY |
| NR2 | MBB_004 | 30° 8.73' N | 87° 31.74' W | - | 98 | 17.1 | 10/10/12 | YES | YOY |
| NR2 | MBB_005 | 30° 8.73' N | 87° 31.74' W | - | 122 | 31.8 | 10/10/12 | NO | YOY |
| NR2 | MBB_006 | 30° 8.73' N | 87° 31.74' W | - | 157 | 74.1 | 10/10/12 | YES | YOY |
| NR2 | MBB_007 | 30° 8.73' N | 87° 31.74' W | - | 132 | 42.9 | 10/10/12 | YES | YOY |
| NR2 | MBB_008 | 30° 8.73' N | 87° 31.74' W | - | 75 | 7.2 | 10/10/12 | YES | YOY |
| NR2 | MBB_009 | 30° 8.73' N | 87° 31.74' W | - | 54 | 2.6 | 10/10/12 | YES | YOY |
| NR2 | MBB_010 | 30° 8.73' N | 87° 31.74' W | - | 70 | 6 | 10/10/12 | YES | YOY |
| NR2 | MBB_011 | 30° 8.73' N | 87° 31.74' W | - | 137 | 49.9 | 10/10/12 | YES | YOY |
| NR2 | MBB_012 | 30° 8.73' N | 87° 31.74' W | - | 93 | 13.8 | 10/10/12 | NO | YOY |
| NR2 | MBB_013 | 30° 8.73' N | 87° 31.74' W | - | 105 | 19.8 | 10/10/12 | YES | YOY |
| NR2 | MBB_014 | 30° 8.73' N | 87° 31.74' W | - | 72 | 6.9 | 10/10/12 | NO | YOY |
| NR2 | MBB_015 | 30° 8.73' N | 87° 31.74' W | - | 81 | 8.9 | 10/10/12 | YES | YOY |
| NR2 | MBB_016 | 30° 8.73' N | 87° 31.74' W | - | 87 | 12.1 | 10/10/12 | YES | YOY |
| NR2 | MBB_017 | 30° 8.73' N | 87° 31.74' W | - | 99 | 17.9 | 10/10/12 | YES | YOY |
| NR2 | MBB_018 | 30° 8.73' N | 87° 31.74' W | - | 97 | 16.9 | 10/10/12 | NO | YOY |
| NR2 | MBB_019 | 30° 8.73' N | 87° 31.74' W | - | 121 | 31 | 10/10/12 | YES | YOY |
| NR2 | MBB_020 | 30° 8.73' N | 87° 31.74' W | - | 127 | 34.8 | 10/10/12 | YES | YOY |
| NR2 | MBB_021 | 30° 8.73' N | 87° 31.74' W | - | 130 | 39.7 | 10/10/12 | YES | YOY |
| NR2 | MBB_022 | 30° 8.73' N | 87° 31.74' W | - | 130 | 39.4 | 10/10/12 | YES | YOY |
| NR2 | MBB_023 | 30° 8.73' N | 87° 31.74' W | - | 120 | 35.7 | 10/10/12 | YES | YOY |
| NR2 | MBB_024 | 30° 8.73' N | 87° 31.74' W | - | 132 | 39.7 | 10/10/12 | YES | YOY |
| NR2 | MBB_025 | 30° 8.73' N | 87° 31.74' W | - | 159 | 68.8 | 10/10/12 | YES | YOY |
| NR2 | MBB_026 | 30° 8.73' N | 87° 31.74' W | - | 188 | 113.7 | 10/10/12 | YES | YOY |
| NR2 | MBB_027 | 30° 8.73' N | 87° 31.74' W | - | 246 | 230.2 | 10/10/12 | YES | YOY |
| PB1 | JC_539 | 27° 59.274' N | 91° 39.547' W | 399 | 496 | 1484 | 5/30/12 | YES | ADULT |
| PB1 | JC_547 | 27° 59.274' N | 91° 39.547' W | 403 | 497 | 1602 | 5/30/12 | YES | ADULT |
| PB1 | JC_553 | 27° 59.274' N | 91° 39.547' W | 568 | 697 | 4732 | 5/30/12 | YES | ADULT |
| PB1 | JC_555 | 27° 59.107' N | 91° 39.181' W | 659 | 814 | 7135 | 5/30/12 | YES | ADULT |
| PB1 | JC_562 | 27° 59.107' N | 91° 39.181' W | 534 | 648 | 3716 | 5/30/12 | YES | ADULT |
| PB1 | JC_567 | 27° 59.107' N | 91° 39.181' W | 610 | 731 | 5548 | 5/30/12 | YES | ADULT |
| PB1 | JC_569 | 27° 59.107' N | 91° 39.181' W | 515 | 629 | 3246 | 5/30/12 | YES | ADULT |
| PB1 | JC_576 | 27° 59.416' N | 91° 39.487' W | 639 | 781 | 6485 | 7/3/12 | YES | ADULT |
| PB1 | JC_581 | 27° 59.416' N | 91° 39.487' W | 470 | 578 | 2494 | 7/3/12 | YES | ADULT |
| PB1 | JC_585 | 27° 59.416' N | 91° 39.487' W | 564 | 693 | 4732 | 7/3/12 | YES | ADULT |
| PB1 | JC_586 | 27° 59.416' N | 91° 39.487' W | 507 | 631 | 2922 | 7/3/12 | YES | ADULT |
| PB1 | JC_590 | 27° 59.416' N | 91° 39.487' W | 552 | 680 | 3876 | 7/3/12 | YES | ADULT |
| PB1 | JC_592 | 27° 59.416' N | 91° 39.487' W | 458 | 568 | 2122 | 7/3/12 | YES | ADULT |
| PB1 | JC_594 | 27° 59.416' N | 91° 39.487' W | 383 | 483 | 1506 | 7/3/12 | YES | ADULT |
| PB1 | JC_600 | 27° 59.416' N | 91° 39.487' W | 526 | 645 | 3190 | 7/3/12 | YES | ADULT |
| PB1 | JC_611 | 27° 59.416' N | 91° 39.487' W | 395 | 487 | 1376 | 7/3/12 | YES | ADULT |
| PB1 | JC_959 | 27° 59.250' N | 91° 39.231' W | 475 | 592 | 2580 | 5/15/13 | YES | ADULT |
| PB1 | JC_966 | 27° 59.250' N | 91° 39.231' W | 449 | 554 | 2070 | 5/15/13 | YES | ADULT |
| PB1 | JC_1116 | 27° 58.815' N | 91° 39.198' W | 437 | 551 | 2290 | 6/23/13 | YES | ADULT |
| PB1 | JC_1118 | 27° 59.436' N | 91° 39.524' W | 648 | 789 | 7645 | 6/24/13 | YES | ADULT |
| PB1 | JC_1119 | 27° 59.436' N | 91° 39.524' W | 597 | 748 | 5255 | 6/24/13 | YES | ADULT |
| PB1 | JC_1213 | 27° 58.991' N | 91° 39.074' W | 531 | 634 | 3125 | 8/15/13 | YES | ADULT |
| PB1 | JC_1216 | 27° 58.991' N | 91° 39.074' W | 564 | 691 | 4335 | 8/15/13 | YES | ADULT |
| PB1 | JC_1218 | 27° 58.991' N | 91° 39.074' W | 451 | 554 | 2470 | 8/15/13 | YES | ADULT |
| PB1 | JC_1219 | 27° 58.991' N | 91° 39.074' W | 616 | 749 | 5155 | 8/15/13 | YES | ADULT |
| PB1 | JC_1221 | 27° 58.991' N | 91° 39.074' W | 652 | 793 | 6675 | 8/15/13 | YES | ADULT |
| PB1 | JC_1222 | 27° 58.991' N | 91° 39.074' W | 523 | 633 | 3210 | 8/15/13 | YES | ADULT |
| PB1 | JC_1224 | 27° 58.991' N | 91° 39.074' W | 506 | 619 | 2900 | 8/15/13 | YES | ADULT |
| PB1 | JC_1226 | 27° 58.991' N | 91° 39.074' W | 505 | 629 | 3345 | 8/15/13 | YES | ADULT |
| PB1 | JC_1229 | 27° 58.991' N | 91° 39.074' W | 420 | 517 | 1685 | 8/15/13 | NO | ADULT |
| PB1 | JC_1230 | 27° 58.991' N | 91° 39.074' W | 482 | 591 | 2280 | 8/15/13 | NO | ADULT |
| PB2 | PC_057 | 30° 11.333' N | 85° 57.023' W | - | 475 | 1470 | 5/25/11 | YES | ADULT |
| PB2 | PC_058 | 30° 11.333' N | 85° 57.023' W | - | 414 | 1050 | 5/25/11 | YES | ADULT |
| PB2 | PC_059 | 30° 11.333' N | 85° 57.023' W | - | 460 | 1370 | 5/25/11 | YES | ADULT |
| PB2 | PC_060 | 30° 11.266' N | 85° 55.502' W | - | 392 | 1060 | 5/25/11 | YES | ADULT |
| PB2 | PC_061 | 30° 11.266' N | 85° 55.502' W | - | 425 | 1090 | 5/25/11 | YES | ADULT |
| PB2 | PC_062 | 30° 11.266' N | 85° 55.502' W | - | 531 | 1180 | 5/25/11 | YES | ADULT |
| PB2 | PC_063 | 30° 11.266' N | 85° 55.502' W | - | 610 | 3670 | 5/25/11 | NO | ADULT |
| PB2 | PC_064 | 30° 02.430' N | 85° 51.799' W | - | 383 | 798 | 6/23/11 | YES | ADULT |
| PB2 | PC_065 | 30° 02.430' N | 85° 51.799' W | - | 386 | 834 | 6/23/11 | YES | ADULT |
| PB2 | PC_066 | 30° 02.430' N | 85° 51.799' W | - | 362 | 864 | 6/23/11 | NO | ADULT |
| PB2 | PC_068 | 30° 02.430' N | 85° 51.799' W | - | 376 | 684 | 6/23/11 | YES | ADULT |
| PB2 | PC_069 | 30° 03.252' N | 85° 52.024' W | - | 437 | 1394 | 6/23/11 | YES | ADULT |
| PB2 | PC_070 | 30° 03.252' N | 85° 52.024' W | - | 310 | 705 | 6/23/11 | YES | ADULT |
| PB2 | PC_071 | 30° 03.252' N | 85° 52.024' W | - | 391 | 825 | 6/23/11 | NO | ADULT |
| PB2 | PC_072 | 30° 03.252' N | 85° 52.024' W | - | 368 | 735 | 6/23/11 | YES | ADULT |
| PB2 | PC_073 | 30° 03.252' N | 85° 52.024' W | - | 404 | 970 | 6/23/11 | NO | ADULT |
| PB2 | PC_074 | 29° 56.327' N | 85° 59.716' W | - | 451 | 1290 | 7/21/11 | YES | ADULT |
| PB2 | PC_075 | 29° 56.327' N | 85° 59.716' W | - | 426 | 960 | 7/21/11 | YES | ADULT |
| PB2 | PC_076 | 29° 56.327' N | 85° 59.716' W | - | 432 | 1103 | 7/21/11 | NO | ADULT |
| PB2 | PC_077 | 29° 55.583' N | 86° 00.435' W | - | 407 | 837 | 7/21/11 | NO | ADULT |
| PB2 | PC_078 | 29° 55.583' N | 86° 00.435' W | - | 440 | 1053 | 7/21/11 | NO | ADULT |
| PB2 | PC_079 | 29° 55.583' N | 86° 00.435' W | - | 355 | 605 | 7/21/11 | YES | ADULT |
| PB2 | PC_080 | 30° 02.462' N | 86° 00.460' W | - | 543 | 2059 | 7/22/11 | YES | ADULT |
| PB2 | PC_081 | 30° 02.462' N | 86° 00.460' W | - | 474 | 1428 | 7/22/11 | NO | ADULT |
| PB2 | PC_082 | 30° 00.604' N | 86° 02.422' W | - | 357 | 643 | 7/22/11 | YES | ADULT |
| PB2 | PC_083 | 30° 00.604' N | 86° 02.422' W | - | 376 | 719 | 7/22/11 | YES | ADULT |
| PB2 | PC_084 | 30° 00.604' N | 86° 02.422' W | - | 447 | 1362 | 7/22/11 | YES | ADULT |
| PB2 | PC_085 | 30° 00.604' N | 86° 02.422' W | - | 516 | 2066 | 7/22/11 | NO | ADULT |
| PB2 | PC_087 | 30° 00.604' N | 86° 02.422' W | - | 400 | 843 | 7/22/11 | YES | ADULT |
| PB2 | PC_088 | 30° 00.604' N | 86° 02.422' W | - | 360 | 695 | 7/22/11 | YES | ADULT |
| PB2 | PC_089 | 30° 00.604' N | 86° 02.422' W | - | 375 | 721 | 7/22/11 | YES | ADULT |
| PB2 | PC_090 | 30° 00.604' N | 86° 02.422' W | - | 370 | 647 | 7/22/11 | YES | ADULT |
| PB2 | PC_093 | 30° 05.187' N | 85° 56.163' W | - | 395 | 877 | 8/3/11 | YES | ADULT |
| PB2 | PC_094 | 30° 05.187' N | 85° 56.163' W | - | 402 | 1044 | 8/3/11 | NO | ADULT |
| PB2 | PC_095 | 30° 04.060' N | 85° 53.789' W | - | 279 | 748 | 8/3/11 | YES | ADULT |
